# The Potential of *Corchorus olitorius* Seeds Buccal Films for Treatment of Recurrent Minor Aphthous Ulcerations in Human Volunteers

**DOI:** 10.3390/molecules27207020

**Published:** 2022-10-18

**Authors:** Nourhan Hisham Shady, Abdullah H. Altemani, Faisal H. Altemani, Sherif A. Maher, Mahmoud A. Elrehany, Entesar Ali Saber, Ahmed M. Badawi, Fatma Mohamed Abd El-Mordy, Nada M. Mohamed, Mohammed A. S. Abourehab, Ahmed M. Sayed, Usama Ramadan Abdelmohsen, Soad A. Mohamad

**Affiliations:** 1 Department of Pharmacognosy, Faculty of Pharmacy, Deraya University, Universities Zone, New Minia 61111, Egypt; 2 Department of Family and Community Medicine, Faculty of Medicine, University of Tabuk, Tabuk 71491, Saudi Arabia; 3 Department of Medical Laboratory Technology, Faculty of Applied Medical Sciences, University of Tabuk, Tabuk 71491, Saudi Arabia; 4 Department of Biochemistry, Faculty of Pharmacy, Deraya University, Universities Zone, New Minia 61111, Egypt; 5 Department of Biochemistry, Faculty of Pharmacy, New Valley University, El Kharga 71511, Egypt; 6 Department of Histology and Cell Biology, Faculty of Medicine, Minia University, Minia 61519, Egypt; 7 Department of Otorhinolaryngology, Faculty of Medicine, Minia University, Minia 61519, Egypt; 8 Department of Pharmacognosy and Medicinal Plants, Faculty of Pharmacy (Girls), Al-Azhar University, Cairo 11754, Egypt; 9 Department of Pharmaceutical Chemistry, Faculty of Pharmacy, Modern University for Technology, and Information (MTI), Cairo 11754, Egypt; 10 Department of Pharmaceutics, College of Pharmacy, Umm Al-Qura University, Makkah 21955, Saudi Arabia; 11 Department of Pharmaceutics and Industrial Pharmacy, College of Pharmacy, Minia University, Minia 61519, Egypt; 12 Department of Pharmacognosy, Faculty of Pharmacy, Nahda University, Beni-Suef 62513, Egypt; 13 Department of Pharmacognosy, Faculty of Pharmacy, Minia University, Minia 61519, Egypt; 14 Department of Pharmaceutics and Clinical Pharmacy, Faculty of Pharmacy, Deraya University, Universities Zone, New Minia 61111, Egypt

**Keywords:** wound healing, *Corchorus*, molecular docking, buccal films, antioxidant

## Abstract

Aphthous ulcers are very common disorders among different age groups and are very noxious and painful. The incidence of aphthous ulcer recurrence is very high and it may even last for a maximum of 6 days and usually, patients cannot stand its pain. This study aims to prepare a buccoadhesive fast dissolving film containing *Corchorus olitorius* seed extract to treat recurrent minor aphthous ulceration (RMAU) in addition to clinical experiments on human volunteers. An excision wound model was used to assess the in vivo wound healing potential of *Corchorus olitorius* L. seed extract, with a focus on wound healing molecular targets such as TGF-, TNF-, and IL-1. In addition, metabolomic profiling using HR-LCMS for the crude extract of *Corchorus olitorius* seeds was explored. Moreover, molecular docking experiments were performed to elucidate the binding confirmation of the isolated compounds with three molecular targets (*TNF-α*, *IL-1β*, and *GSK3*). Additionally, the in vitro antioxidant potential of *C. olitorius* seed extract using both H_2_O_2_ and superoxide radical scavenging activity was examined. Clinical experiments on human volunteers revealed the efficiency of the prepared *C. olitorius* seeds buccal fast dissolving film (CoBFDF) in relieving pain and wound healing of RMAU. Moreover, the wound healing results revealed that *C. olitorius* seed extract enhanced wound closure rates (*p* ≤ 0.001), elevated *TGF-β* levels and significantly downregulated *TNF-α* and *IL-1β* in comparison to the Mebo-treated group. The phenotypical results were supported by biochemical and histopathological findings, while metabolomic profiling using HR-LCMS for the crude extract of *Corchorus olitorius* seeds yielded a total of 21 compounds belonging to diverse chemical classes. Finally, this study highlights the potential of *C. olitorius* seed extract in wound repair uncovering the most probable mechanisms of action using in silico analysis.

## 1. Introduction

Buccal and mucoadhesive films are among the dosage forms that adhere well to different patient categories. Adults, children, or the elderly can easily self-administer buccoadhesive film and manage their doses [1,2]. The casting of hydrophilic polymer dispersions is the most common method of buccoadhesive film preparation [1,2,3]. Polymers such as polyvinyl alcohol, hydroxypropyl methylcellulose, chitosan, and Eudragit are among these polymers. The Eudragit-based fast-dissolving film is more advantageous than other hydrophilic polymers types for its adherence to the oral mucosa and forming a clear film [4]. Aphthous ulcers are very common disorders among different age groups which are very noxious and painful. The incidence of aphthous ulcer recurrence is very high and may even last for a maximum of 6 days, patients cannot stand its pain [5,6]. Diet supplements nowadays can solve many health problems safely and economically. *Corchorus* (jute), belongs to the family *Tiliaceae*, it is a genus of annual herbs which involves about 50–60 species distributed in tropical, subtropical, and warm temperate regions throughout the world. The origin of *Corchorus olitorius* L., which is commonly known as Molokhia, and most other species, is Africa [7]. *Corchorus olitorius* L., is a leafy, green, summer vegetable. In addition to being a medicinal plant, it is also a famous traditional dish in the Middle East and different parts of Asia and Africa [8]. *Corchorus olitorius* L. is a tall, erect, annual herb, up to 2.4 m highest, and is well-branched. The leaves are simple, alternate, ovate-lanceolate to lanceolate, with finely serrated or lobed margins [9]. The plant bears yellow flowers that are hermaphrodite and pollinated by insects. The flowers are small about 2–3 cm in diameter, with five petals. The fruits are multi-seeded capsules, elongated, straight or curved, dehiscent, and divided into transverse sections through five valves [10]. Different parts of *Corchorus olitorius* L., have been used traditionally as a herbal medicine to relieve pain, aches, chronic cystitis, dysentery, enteritis, and pectoral pains [11]. *Corchorus olitorius* leaves are used in cases of gonorrhea, chronic cystitis, fever, and tumors, while consumption of the seeds has been observed to be demulcent, diuretic, purgative, and used in cases of cardiac diseases such as heart failure due to its high content of cardiac glycosides (cardenolides) [7,12]. *Corchorus olitorius* L., has been found to have several pharmacological activities as its leaves have an anti-inflammatory, antihypertensive effect, [13], antidiabetic activities by showing hypoglycemic and hypolipidemic effects [14], antioxidant and wound healing effect [15], gastroprotective activity [16], and antiviral activity in the treatment of measles [17], while the aerial parts have antitumor activities against melanoma, leukemia, and osteosarcoma [18]. The plant also shows significant antimicrobial and antifungal activities [19]. Phytochemical investigations of *Corchorus* *olitorius* L., seeds revealed the presence of cardiac glycosides such as coroloside, veticoside, erysimoside, helveticoside, corchoriside A, corchoriside B, strophanthidol, strophanthidin, evonoside, chorchorusoside A–E [20], in addition to three new cardenolide glycosides cannogenol 3-*O*-*β*-d-glucopyranosyl-(1→4)-*O*-*β*-d-boivinopyranoside, periplogenin 3-*O*-*β*-d-glucopyranosyl-(1→4)-*O*-*β*-d-digitoxopyranoside and digitoxigenin 3-*O*-*β*-d-glucopyranosyl-(1→6)-*O*-*β*-d-glucopyranosyl-(1→4)-*O*-*β*-d-digitoxopyranoside [12]. On the other hand, triterpenes, intones, steroids, acidic polysaccharide which are rich in uronic acid and consisting of rhamnose, glucose, galacturonic acid, and glucuronic were reported in *Corchorus* *olitorius* L., leaves [20,21]. Moreover, the *Corchorus* *olitorius* L., the root was found to contain corosin, β-sitosterol, and triterpene [20,22]. The nutraceutical effect of *Corchorus olitorius* L. as a wound healing agent was previously reported [15]. This provoked us to investigate the wound healing and antioxidant potential of *Corchorus olitorius seeds* supported by in vitro and in vivo studies exploring the histopathological consequences following skin injury and application of the seed extract. Furthermore, studying the metabolomic profiling of *Corchorus olitorius* seed extract to reveal the chemical constituents responsible for its wound healing potential. Additionally, docking studies were carried out for our compounds. The aim of this study is to prepare a buccoadhesive fast-dissolving film containing *Corchorus olitorius* seed extract for the treatment of recurrent minor aphthous ulcerations (RMAU). The film was characterized in vitro for appearance, size and weight, disintegration time and surface morphology, in addition to clinical experiments on human volunteers. The clinical trial was registered on Clincaltrials.gov under the number (ClinicalTrials.gov Identifier: NCT05392842). The study was intervention randomized and double blinded to compare the new intervention with a placebo in ulcer size, inflammation degree and pain scoring. The main aims of the trial were the safety and effectiveness of the new intervention.

## 2. Results

### 2.1. Metabolomic Profiling of Corchorus olitorius Seeds

The metabolomic profiling using HR-LCMS for the crude extract of *Corchorus olitorius* seeds was carried out to identify the chemical compounds responsible for the wound healing potential. The dereplicated compounds of the *Corchorus olitorius* seeds as shown in Table 1 belonged to different chemical classes. Based on the compounds that were discovered, identification of the compounds was carried out using HR-ESIMS and comparison with the data reported in the literature such a corchorifatty acid C that was previously isolated from the leaves of *Corchorus olitorius* and exhibited an inhibitory effect on lipopolysaccharide-induced NO production, since high levels of NO production cause inflammation, immunological response, so corchorifatty acid C (**1**) was expected to have anti-inflammatory potential [23]; digitoxigenin boivinoside (**2**) was previously isolated from the seeds of *Corchorus olitorius* [24]; 4,7-Dihydroxycoumarin (**3**) has been identified from seeds and leaves of *Corchorus olitorius* [25]; 9-Hydroxy-10-undecenoic acid (**4**) was reported to have a neuroprotective effect by exhibiting potent NGF secretion in C6 cell [23]; corchorifatty acid B (**5**) previously detected in the leaves of *Corchorus olitorius* was found to inhibit the LPS-induced NO production in cultured mouse peritoneal macrophages [23]; corchorifatty acid F (**6)** was previously isolated from leaves of *Corchorus olitorius*, and was supposed to have anti-inflammatory potential due to inhibition of the LPS-induced NO production [23]; corchoionoside C (**7**) was reported in leaves of *Corchorus olitorius* [26]; corchoionoside A and B (**8**–**9**) were reported to inhibit the histamine release from rat peritoneal cells induced by antigen antibody reaction [27]; corchoroside B (**10**) [24], trachelosperogenin A (**11**) was reported to have antioxidant potential [28]; corchoroside A (**12**) was previously isolated from the seeds of *Corchorus olitorius* [29], corchorosol A (**13**) was isolated from the seeds of *Corchorus olitorius*; corchoruside B (**14)** is a flavonol glycoside isolated from the seeds of *Corchorus olitorius* [30]; coroloside (**15**) was isolated from the seeds of *Corchorus olitorius*, applied for heart failure treatment [12]; corchorusoside B and olitoriside (**16**–**17**) [31,32], corchorusoside D and corchorusoside A (**18**–**19**) [31], olitoriusin (**20**) [32], corchorusoside E (**21**) were previously isolated from the seeds of *Corchorus olitorius* [31].

### 2.2. In Vitro Antioxidant Activity

#### 2.2.1. Hydrogen Peroxide Scavenging Activity

**Figure 1:**
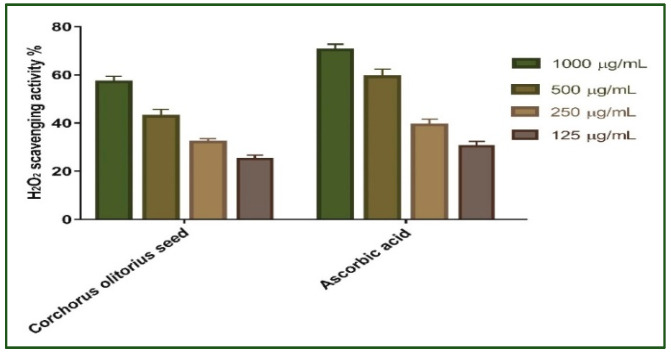
H_2_O_2_ radical scavenging activity of *Corchorus olitorius* seed extract at different concentrations (1000, 500, 250, and 125 µg/mL). Bars represent mean ± standard deviation (SD). Significant difference between groups is analyzed by a two-way ANOVA test after normalization of variables by the Shapiro–Wilk test.

Antioxidants are thought to manage wound oxidative stress and hence speed up the wound healing process. They play a critical role in controlling the damage of biological components such as DNA, protein, lipids, and body tissue that may sustain the presence of reactive species [33]. The antioxidant activity of *Corchorus olitorius* seed extract as a scavenger potential against H_2_O_2_ was investigated in this study. The maximal hydrogen peroxide radical scavenging activity of *Corchorus olitorius* seed extract was 57.14 percent at 1000 µg/mL concentration, according to the data. *Corchorus olitorius* seed extract suppressed the formation of hydrogen peroxide radicals in a dose-dependent manner, demonstrating a consistent antioxidant activity with IC_50_ of 174.7 μg/mL (Figure 1) and was compared with standard ascorbic acid (IC_50_ = 182.1 μg/mL). High levels of reactive oxygen species (ROS) in the wound site can promote collagen breakdown and hence the destruction of the extracellular matrix (ECM). When the ECM is destroyed, processes such as angiogenesis and re-epithelization, which are crucial for wounds to heal are reduced [27,34]. Moreover, elevated ROS can induce inflammation and increase pro-inflammatory cytokines and hence prolong inflammation [35]. The *Corchorus olitorius* seed extract’s SOD activity and H_2_O_2_ scavenging activity, both of which are attributable to its antioxidant activity, can remove ROS and so improve its ability to heal wounds. This antioxidant potential may be attributed to the phenolic content of the extract.

#### 2.2.2. Superoxide Radical Scavenging Activity

**Figure 2:**
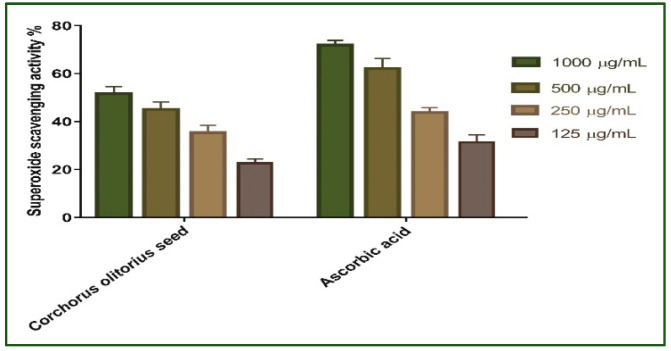
Superoxide radical scavenging activity of *Corchorus olitorius* seed extract at different concentrations (1000, 500, 250, and 125 µg/mL). Bars represent mean ± SD (standard deviation). Significant difference between groups is analyzed by a Two-way ANOVA test after normalization of variables by the Shapiro Wilk test.

Redox signaling and enhanced oxidative stress play an important role in normal wound healing by encouraging hemostasis, inflammation, angiogenesis, granulation tissue creation, wound closure, and extracellular matrix development and maturation [36]. As a result, the superoxide scavenging activity of *Corchorus olitorius* seed extract was evaluated. The results revealed that the scavenging impact of the standard and extract rises with concentration (Figure 2); in addition, *Corchorus olitorius* seed extract exhibits the maximum superoxide radical scavenging activity. *Corchorus olitorius* seed extract has 51.61% superoxide scavenging efficacy at 1000 μg/mL concentration. The concentration of *Corchorus olitorius* seed extract needed for 50% inhibition (IC_50_) was found to be 148.9 µg/mL whereas 157.7 μg/mL was needed for ascorbic acid.

### 2.3. Wound Healing Activity

#### 2.3.1. Wound Closure Rate Estimation

Wound healing is a complex process of repairing tissue structures in injured tissue as closely as possible to their natural presence [36,37]. Dermal wound repair contains three phases: an inflammatory process owing to pro-inflammatory-mediator secretion and immune system suppression, a proliferative phase via the proliferation of fibroblasts, collagen growth, and fresh blood vessel development as well as a remodeling phase that covers regeneration and injured tissue repair [38,39]. Therefore, drugs that could accelerate wound repair with a potential input in all phases of the process will be required for efficient therapy, specifically those with lower costs and fewer side effects.

**Figure 3:**
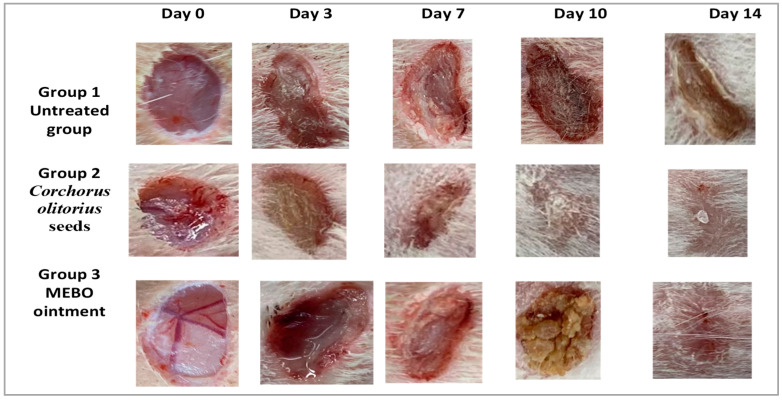
The activity of Corchorus olitorius seed extract and Mebo on excisional wounds on days 0, 3, 7, 10, and 14 post-wounding in the wound of adult male New Zealand Dutch strain albino rabbits, group 1: untreated (bare group), group 2: *Corchorus olitorius* seed extract-treated group and group 3: Mebo-treated group as a positive control group.

Our results showed that the flow of wound closure in all experimental groups increased in a time-dependent manner. On the third day post-injury, the wound closure rate was around 9 to 19% in each group, being the smallest in the untreated group and the highest in the treated ones, with no significant difference (*p* > 0.001) between groups. On the seventh day after treatment, the wound closure in the *Corchorus olitorius* seed extract-treated group reached 50%, which appeared to be significantly higher (*p* ≤ 0.001) than the corresponding untreated group (Figure 3).

**Figure 4:**
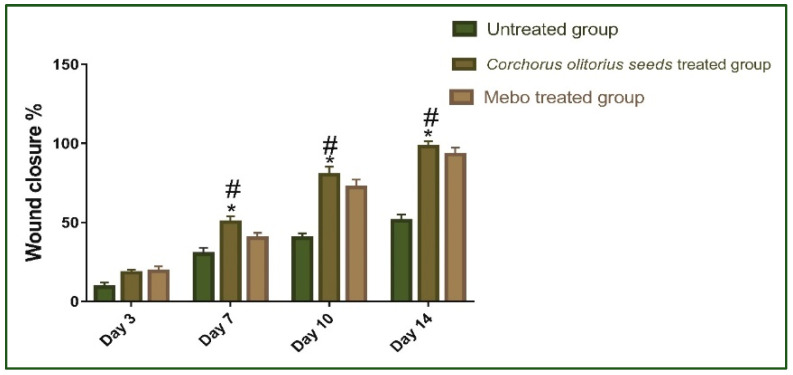
Wound closure rates in all tested groups (group 1: untreated group bare control (*n* = 8), group 2: *Corchorus olitorius seed extract*-treated group (*n* = 8), group 3: Mebo-treated group (*n* = 8)) over time post-injury (0, 3, 7, 10, and 14 days). Significant difference between groups is analyzed by a two-way ANOVA test after normalization of variables by the Shapiro–Wilk test. Data were expressed as mean ± SD. * *p* ≤ 0.001 compared with those of the untreated group on the respective day and # *p* ≤ 0.001 compared with those of the Mebo group on the respective day.

In addition, the *Corchorus olitorius* seed extract-treated group also showed faster-wound closure rates compared to those of the Mebo-treated group (40%) (*p* ≤ 0.001). The wound closure rates of the *Corchorus olitorius* seed extract-treated group (80%) were again significantly higher (*p* ≤ 0.001) than the untreated group (40%) on the 10th day post-burn. On the 14th day post-burn, the wounds in the *Corchorus olitorius* seed extract-treated group were completely healed and the wound closure reached 98% in the *Corchorus olitorius* seed extract-treated group and 93% in the Mebo-treated group (Figure 4). In addition, the accelerated wound closure rate was outstanding in *Corchorus olitorius* seed extract-treated wounds. Wound closure can be represented as the centripetal flow of the edges of a full-thickness wound to aid the closure of the wound tissue [40,41,42]. Wound closure is thus an indicator of re-epithelialization, granulation, angiogenesis, fibroblast proliferation, keratinocyte differentiation, and proliferation [42].

#### 2.3.2. Effect of *Corchorus olitorius* Seed Extract on Expression of *TGF-β*, *TNF-α*, *IL-1β*

**Figure 5:**
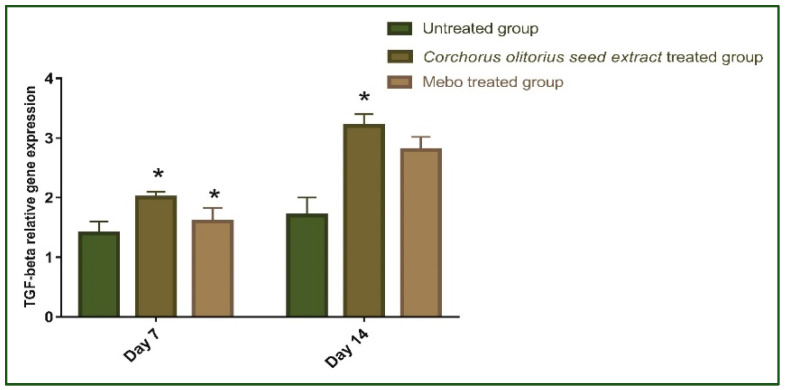
Gene expression in wound tissues for rabbits of different groups via quantitative RT-PCR. It was used to evaluate the gene expression in wound tissues for rabbits of different groups. Data represent fold change relative to the normal control group expression after normalization to glyceraldehyde 3-phosphate dehydrogenase (*GAPDH*). Bars represent mean ±SD. Significant difference between groups is analyzed by a two-way ANOVA test, where: * *p* ≤ 0.001 compared with those of the untreated group on the respective day.

Figure 5 depicts the mRNA expression of *TGF-β* following excisional wound therapy with *Corchorus olitorius* seed extract and Mebo. TGF-β relative mRNA expression in skin tissues was substantially higher in *Corchorus olitorius* seed extract-treated wounds for 7 or 14 days compared to the untreated group (*p* ≤ 0.001). The relative expression of *Corchorus olitorius* seed extract-treated wounds, on the other hand, showed a significant rise in the marker’s expression as compared to the Mebo-treated group. Wound-healing processes require complex interactions between cells as well as numerous growth factors [43], where the *TGF-β* hits the most crucial part throughout all phases of wound healing. During the hemostasis and inflammation phase, the *TGF-β* recruits and activates inflammatory cells, covering neutrophils and macrophages, whereas, in the proliferative phase, it creates multiple cellular responses having re-epithelialization, angiogenesis, granulation tissue development, and extracellular matrix deposition [44]. It stimulates fibroblasts to multiply and differentiate into myofibroblasts that participate in wound closure in the remodeling phase [45,46,47]. Chronic, non-healing wounds generally produce a failure of *TGF-β1* warning, while Feinberg and his co-worker [48] have declared that *TGF-β1* delivers an inhibitory effect on the expression of collagenases, which impair collagen and extracellular matrix. These notes are coherent with the above measurements, which established that *Corchorus olitorius* seed extract enhanced *TGF-β1* expression and hence recovered wound healing. The mRNA expression investigation of the wound tissues produced an increment in *TGF-β1* levels in *Corchorus olitorius* seed extract-treated wound tissues related to the untreated wound tissues. This may signify that *Corchorus olitorius* seed extract encouraged the expression of *TGF-β1* in the wound tissues.

**Figure 6:**
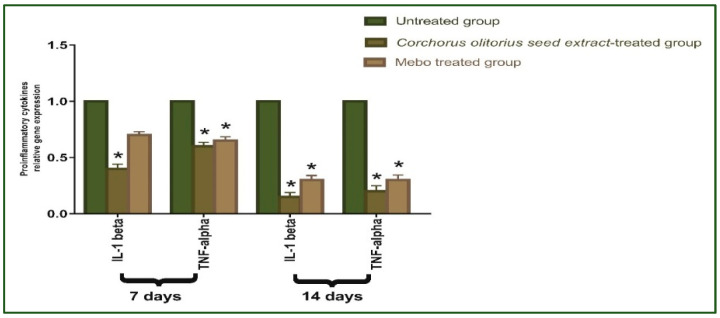
Gene expression in wound tissues for rabbits of different groups. Quantitative RT-PCR was used to evaluate the gene expression in wound tissues for rabbits of different groups. Data represent fold change relative to the normal control group expression after normalization to glyceraldehyde 3-phosphate dehydrogenase (*GAPDH*). Bars represent mean ± SD. Significant difference between groups is analyzed by a two-way ANOVA test, where: * *p* ≤ 0.001 compared with those of the untreated group on the respective days.

As shown in Figure 6, the mRNA expression of *TNF-α* and *IL-1β* was illustrated. Analysis of mRNA expression of full-thickness wound samples on day 7 post-injury revealed that the activity of the inflammatory markers *TNF-α* and IL-1β was significantly down-regulated in wounds treated with *Corchorus olitorius* seed extract or Mebo compared to the untreated wounds. However, wounded rabbits treated with *Corchorus olitorius* seed extract displayed a significantly much more reduction in the inflammatory markers (*TNF-α*, and *IL-1β*) when compared to the Mebo-treated group. Moreover, *Corchorus olitorius* seed extract treatment or Mebo treatment for 14 days showed a significantly dramatic decrease in *TNF-α* and *IL-1β* mRNA expression when compared to the untreated group at (*p* ≤ 0.001). The expression of *TNF-α* and *IL-1β* in *Corchorus olitorius* seed extract-treated wounds was markedly lower than in the Mebo-treated group [1]. Suitable expression of pro-inflammatory cytokines (*IL-1β* and *TNF-α*) is necessary to recruit neutrophils and exclude bacteria, and other contamination from the wound site and is recognized as the dynamic inducer of metalloproteinase (MMP) synthesis in inflammatory and fibroblasts cells. In wound healing, the MMP degrades and removes damaged extra-cellular matrixes (ECM) to aid wound repair [49]. However, a lengthy duration of the inflammatory phase leads to a problem in the healing process and these cytokines and proteinase damage the tissue and lead to the development of chronic wounds. *TNF-α* is one of the growth factors secreted from macrophages, which mixes with *IL-1β* to increase and suppress, respectively, collagen production and fibroblast proliferation [50]. The *TNF-α* stimulates NF-κB, which in turn promotes gene expression of a plethora of pro-inflammatory cytokines including *TNF-α* itself and proteases, such as MMP, to free soluble *TNF-α* and potentiate the effects of this inflammatory cytokine [43]. So, suppressing inflammatory cytokines (*TNF-α*, and *IL-1β)* by *Corchorus olitorius* seed extract can inhibit continued inflammation and hence avoid impaired wound repair. These results suggested that *Corchorus olitorius* seed extract could accelerate the switching process from inflammatory to anti-inflammatory responses, which afterward increase healing.

#### 2.3.3. Histopathological Study

**Figure 7:**
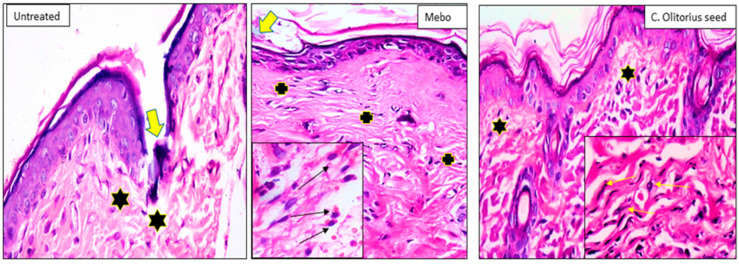
Wounded skin 7 days after incision; [untreated group] (*n* = 8), showing the wound filled with blood clots (thick arrow) and the underlying sloughed granulation tissue with compact and disorganized collagen bundles (stars). [Mebo-treated group] (*n*= 8), showing scar tissue blocking the wound (thick arrow), the underlying dermal matrix showing inflammatory cellular infiltration mainly macrophages with acidophilic cytoplasm (black arrows in the inset) and coarse wavy collagen bundles packing the defect resembling that of the adjacent normal dermis (crosses). [*C. olitorius* seed-treated group] (*n* = 8), Showing marked re-epithelization and the underlying regenerated papillary dermal tissue showing fine interlacing collagen bundles (stars), and in the reticular dermis appear as coarse wavy bundles with numerous fibroblasts (yellow arrows in the inset). (H & E stain ×200 and 400).

Seven days after treatment, group 1 (untreated group) showed a normal edge of the wound with normal architecture, having epidermis, well-formed dermal collagen bundles, hair follicles, and sebaceous glands. The wound is filled with blood clots, sloughed granulation tissue with collagen fibers compactly organized in an irregular pattern, extravasated RBCs, and inflammatory cellular infiltration. The striated muscle showed necrotic myofiber in the deepest part of the wound. In group 2 (*Corchorus olitorius* seed extract-treated group), the blood clot observed over the wound was still apparent, incomplete marked re-epithelization and the granulation tissue filling the defect from below was mainly cellular. Disorganized dense collagen with fibers appeared compactly arranged in an abnormal pattern resulting in distinct scarring in comparison to other treated groups. Group 3 (Mebo-treated group), scar tissue closing the wound and creeping of epidermal cells at wound edges were marked with a partial re-epithelization. A marked inflammatory cellular infiltration (mainly of macrophages) and collagen fibers were packing the defect in a reticular pattern with a distance in between approximately resembling that of the neighboring natural dermis [51]. The reticular dermis contained the usual active, lengthened, and spindle-shaped fibroblasts with basophilic cytoplasm and open-face oval nuclei as shown in Figure 7.

**Figure 8:**
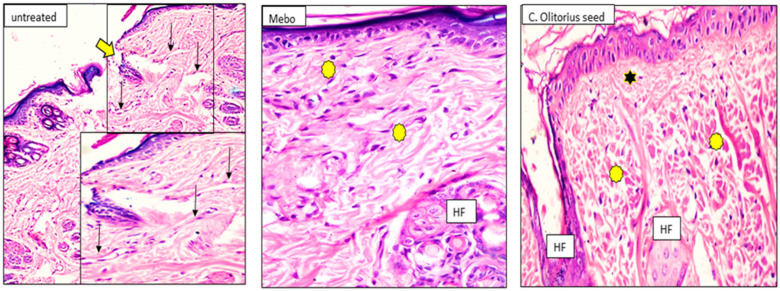
Wounded skin 14 days after incision; [untreated group] (*n* = 8), showing the wide wound area (thick arrow) and the dermis underneath showing heavy inflammatory cellular infiltration and the blood vessels appear dilated and congested (arrows). [Mebo-treated group] (*n* = 8), showing typical epidermis and the underlying dermis with coarse and compact collagen bundles arranged in different directions (asterisks). Newly formed hair follicles (HF). [*C. olitorius* seed-treated group] (*n* = 8), showing typical stratified squamous keratinized epithelium and the collagen in the papillary dermis appearing as fine interlacing bundles (stars), and in the reticular layer appearing as coarse and wavy bundles (asterisks). Notice the newly formed hair follicles (HF). (H & E stain ×200 and 400).

Fourteen days after treatment, group 1 (untreated group) developed a larger wound area that was packed with a heavy coat of granulation material, which was composed of several layers of connective tissue cells in an acidophilic matrix and overlying fat inflammatory cellular infiltration. The dermis was composed of confused, weak collagen with marked neovascularization. Group 2 (*Corchorus olitorius* seed extract-treated group) showed contracted scar tissue blocking the wound and the epidermis appeared formed of only 1–3 rows of epithelial cells. The granulation tissue from below was mainly cellular and populated with fibroblasts, while the reticular layer contained disorganized dense compactly arranged collagen fibers. In group 3 (Mebo-treated groups), the skin tissue presented as more or less normal with a normal stratified squamous keratinized epithelium. Weak scar tissue was spreading into the dermis. The dermal matrix showed many hair follicles, blood capillaries, and a deficiency of inflammatory cellular infiltration. The collagen bundles in the papillary dermis were presented as fine interlacing bundles, and the reticular dermis was presented as coarse wavy bundles formed in various paths as shown in Figure 8.

### 2.4. Corchorus Olitorius Seeds Buccal Films for Treatment of Recurrent Minor Aphthous Ulcerations

**Figure 9:**
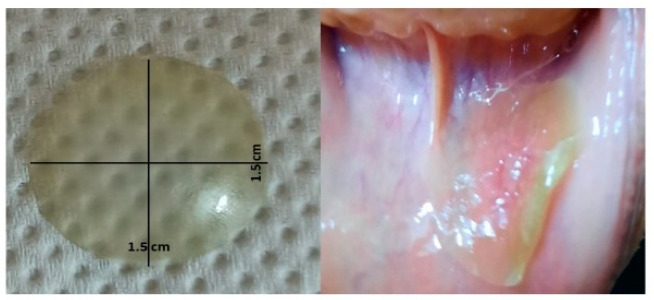
Film dimensions and its adhesion to the buccal mucosal membrane.

The prepared CoBFDF visually exhibited a lack of defects. It was translucent, slightly green and with no marks of cracking, and had a double face strong adhesion property (Figure 9). The prepared films exhibited average weight ranging from 20.3 ± 0.2 to 45.8 ± 2.5 mg and thickness between 0.05 ± 0.02 and 0.28 ± 0.02 mm. The films displayed pH values ranging from 6.8 ± 0.1 to 7.2 ± 0.1.

**Figure 10:**
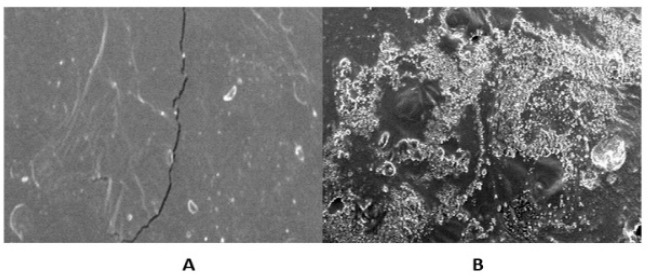
Scanning electron microscopic of the plain film (**A**), and drug-loaded film (**B**).

The in vitro disintegration time of the prepared films varied from 20 ± 3 to 70 ± 5 s. The entire morphology of the film showed significant differences before and after the addition of the Corchorus extract. The prepared Corchorus BFDF and the corresponding free one were scanned at a magnification power of 100 µm. The free film showed soft surfaces, not cluttered, without slanting incisions, Figure 10A, while the Corchorus BFDF showed an irregular cluttered, porous surface, Figure 10B.

The prepared buccoadhesive films had a bright appearance, size, and weight. The estimated weight variation was acceptable revealing the even distribution of the ingredients and the suitability of the applied formulation procedures. The thickness of the prepared films showed a narrow range that was suitable for ease of buccal application affording convenience upon application in the oral region [1,3]. The disintegration time is very suitable for the patient to reduce pain and compatible with the polymer’s ratios. The rapid disintegration time depends on the high percent of HPMC relative to PVA [52].

#### The Clinical Assessment

The clinical examination of patients after four days of following revealed a significant difference between the two arms of the study. Pain score, ulcer size, and erythema were completely different between the two groups. The efficiency index confirmed the rate of change before and after the administration of supplemental buccal films. Table 2 shows the sociodemographic analysis of the two groups that revealed no statistical difference between the participants of the two groups in age, sex, residence, level of education, and the onset of ulcers. All participants had no previous history of chronic diseases and agreed to share and be photographed in addition to signing a consent form. The clinical assessment of treatment outcomes (pain score, ulcer size, and erythema) was evaluated in the first visit, after 3 days, and after 6 days, for best evaluation of improvement with time. Zero-time evaluation of the two groups showed no significant difference between the intervention and control group in pain score, ulcer size, and erythema, *p*-value 0.74, 0.71, and 0.81, respectively, as shown in Table 3. This indicates no statistical differences at the start of the study. Treatment outcomes were significantly different after 3 days of treatment with *p*-values of 0.042, 0.0052, and 0.034 for pain score, ulcer size, and erythema, respectively, showing the potential of the new intervention as shown in Table 4. The indices of efficiency showed a remarkable difference between the groups of study after 6 days of treatment as illustrated in Table 5. The intervention group (*Corchorus olitorius* seeds BFDF) showed a remarkable improvement (75%) in the efficiency of reduction of pain intensity at the end of treatment, whereas the control group showed moderate improvement (45%) in the same period.

**Figure 11:**
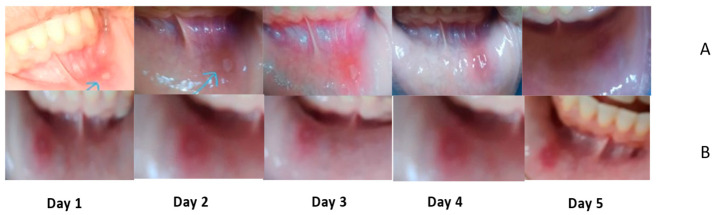
Daily progress in RAMU healing in (**A**) intervention group, (**B**) control group.

The efficiency of CoBFDF in relieving pain and wound healing is clear in Figure 11. which shows the daily progress of RAMU.

### 2.5. Wound Healing In-Silico Molecular Modeling

**Figure 12:**
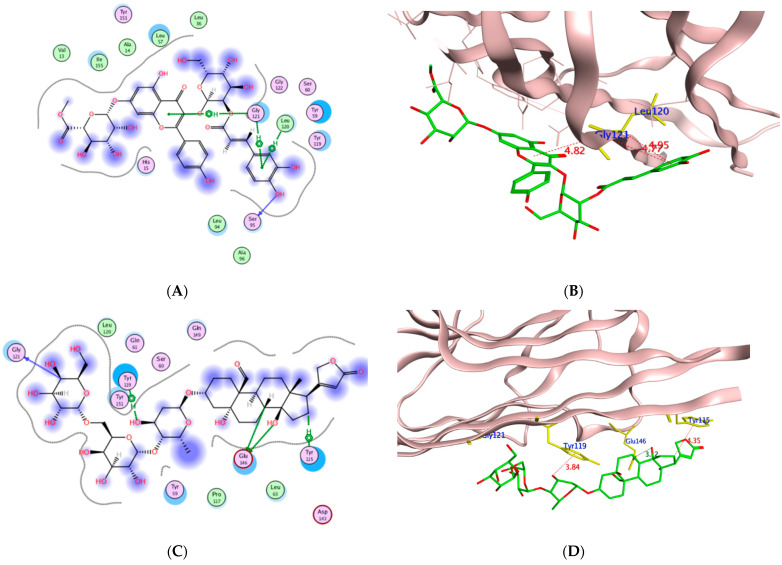

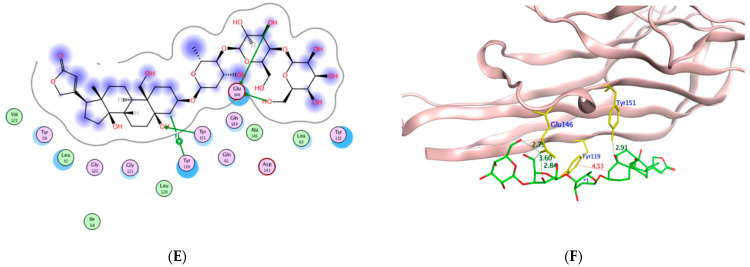
The 2D and 3D molecular docking presentation of the possible binding conformations of **19** (**A**,**B**), **20** (**C**,**D**), and **21** (**E**,**F**) in the *TNF-α* active site using PDB 2AZ5. The interacting residues are highlighted in the yellow stick model with H-bonds and H-pi interactions are presented as green and red dotted lines, respectively, having their distances illustrated in Å.

**Figure 13:**
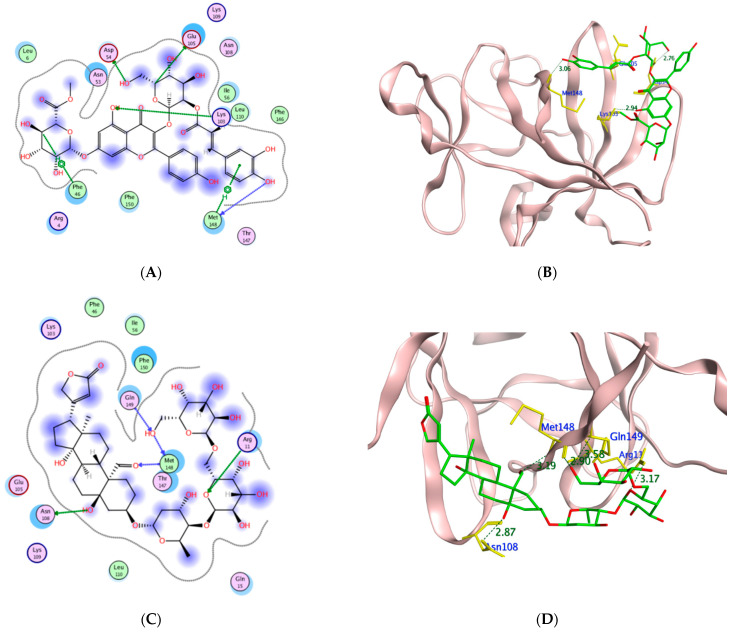

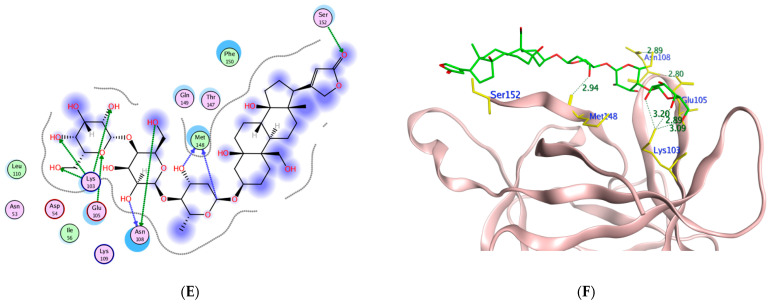
The 2D and 3D molecular docking presentation of the possible binding conformations of **19** (**A**,**B**), **20** (**C**,**D**), and **21** (**E**,**F**) in the *IL-1β* binding site using PDB 6Y8M. The interacting residues are highlighted in the yellow stick model with H-bonds and H-pi interactions are presented as green and red dotted lines, respectively, having their distances illustrated in Å.

**Figure 14:**
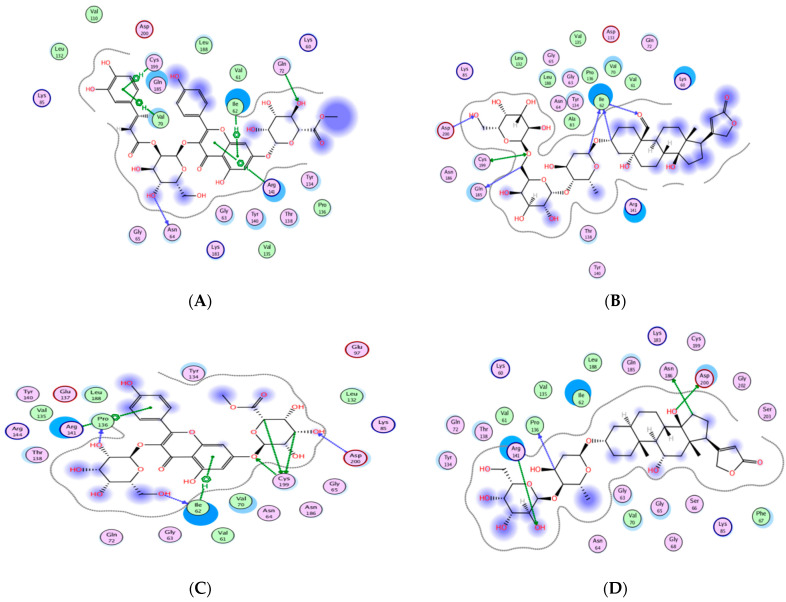
The 2D presentations of the molecular interaction of **14** (**A**), **16** (**B**), **19** (**C**), and **20** (**D**) with *GSK3* using PDB 1Q5K.

**Figure 15:**
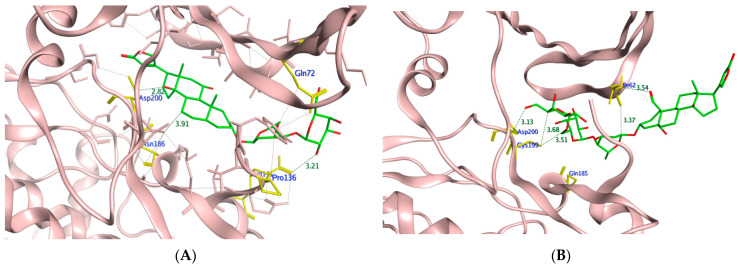
The 3D presentations of the molecular interaction of **16** (**A**) and **20** (**B**) with *GSK3* using PDB 1Q5K are illustrated as a green stick model. The interacting amino acids of the binding domain are shown in yellow with the formed H-bonds and H-arene appearing as a green and red dotted line, respectively, with their corresponding distance in Å.

To assess the potential of the isolated compounds **1–21** to support the wound healing process and anti-inflammatory effect; *TNF-α*, *IL-1β*, and glycogen synthase kinase 3*β* (*GSK3*) were selected for their in-silico evaluation. The regulatory enzyme *GSK3* is a crucial member of the highly ordered wound healing machinery whose inhibition accelerates the *β*-catenin-dependent Wnt signaling pathway which ultimately enhances the wound healing process [52,53]. It is worth mentioning that the Wnt glycoproteins assist in controlling the proliferation process, differentiation, and cell fate [54]. The dereplicated compounds **1**–**21** binding orientation and energy were visualized through molecular docking using PDB 2AZ5, 6Y8M, and 1Q5K for *TNF-α*, *IL-1β*, and *GSK3*, respectively [54,55,56]. The docking protocol was validated before commencing compound docking to ensure the reliability of the results by re-docking the co-crystallized ligands giving RMSD of 2.0, 1.82, 0.67 Å for *TNF-α*, *IL-1β* and GSK-3, respectively. The molecular docking results are illustrated in Table 6 and Table 7 and Figure 12, Figure 13, Figure 14 and Figure 15.

From the achieved results of *TNF-α* molecular docking, it was observed that compounds **10** and **14–21** showed better binding energy than the co-crystallized ligand 307 giving a range of −7.38 to −9.37 kcal/mol compared to −7.16 kcal/mol for 307. Moreover, it was found that **15** of the **21** isolated compounds managed to interact with the crucial Tyr119 by either hydrogen or hydrogen-pi interaction of an approximate length of 3.0 Å and 4.0 Å, respectively. Based on the reported amino acid sequence of *TNF-α*, Tyr119 is essential to accommodate the dimmer form of this cytokine which in turn is essential for substrate binding [57,58]. Additionally, several compounds are bound to other amino acids of the active site of *TNF-α* such as Leu120, Gly121, and Tyr151 for acceptable positioning inside the pocket. The best compounds to interact with *TNF-α* resulting in the proposed inhibitory activity were the highly hydroxylated compounds **19–21** suggesting the significance of the polar groups’ presence to bind to the active site residues. Their binding energy ranged from −8.83 to −9.37 kcal/mol out of which **20–21** formed H-pi interactions with Tyr119. Furthermore, corchorusoside E **21** formed H-bonds with another tyrosine residue of the active site Tyr151 that distanced 2.91 Å. On the other hand, corchorusoside A **19** and olitoriusin **20** established H-pi and H- bonds with the active site Gly121, respectively. Considering the in-silico *IL-1β* binding orientation of the isolated compounds **1–21**, all compounds showed better binding energy than its co-crystallized ligand SX2203 as illustrated in Table 6. Similar to *TNF-α*, compounds **19–21** showed the best binding energy ranging from −9.17 to −9.74 kcal/mol compared to −4.77 kcal/mol of SX2203 with their binding conformations illustrated in Figure 13. As retrieved from the reported protein sequence of *IL-1β*, it possesses two binding sites; A and B to interact with its receptor and commence the inflammatory cascade. The crucial residues of site A were Arg11 and Gln15, while for site B they were Arg4, Leu6, Phe46, Gln48, Glu51, Asn53, Ile56, Lys93, Glu105, Asn108, and Phe150 [55,57]. As depicted from the docking results, corchoroside B **10** bound through H-bonds with both Arg11 and Gln15 of *IL-1β* site A with an approximate distance of 3.0 Å. However, most of the compounds interacted with amino acid residues of site B with either H-bonds or pi-H interactions such as Phe46, Asn53, Glu105, Asn108, and Phe150. In the same context, most of the compounds shared the ability to bind with Lys103 and Met148 with SX2203 which assisted in evaluating their inhibitory potential.

By inspecting the molecular docking results of *GSK3*; the 4,7-dihydroxy coumarin **3** showed the highest binding energy while corchorusoside B **16** had the lowest energy with −5.39 and −11.49 Kcal/mol, respectively. Furthermore, it was noticed that the poly-hydroxylated derivatives **14–21** showed much lower binding energy than the other derivatives, indicating the importance of the compound hydrophilicity in forming several hydrogen bonds with the enzyme binding site. Moreover, through recognizing the crucial amino acids of the *GSK3* activation domain [59], it was found that many H-bonds were formed between Lys85 and compounds **2,4,8–9,11,** and **18,** while analogs **13** and **19** managed to bind to Asn67 and Asn64, respectively, through H- bonding. Similarly, Val135 acted as H-bond donor to **3, 6, 7,** and **10;** on the other hand, Val70 fashioned an H-arene interaction with **3** and **19** through their aromatic rings.

To gain further insight into the molecular orientation of corchoruside B **14**, corchorusoside B **16**, corchorusoside A **19,** and olitoriusin **20** with the best binding energy, their 2D and 3D of **16** and **20** interactions with *GSK3* protein are illustrated in Figure 14 and Figure 15, respectively.

## 3. Materials and Methods

### 3.1. Plant Material

The seeds of *Corchorus olitorius* were collected in February 2021, from the Minya area, Egypt. A voucher specimen (Corc-2-2021) was archived at Pharmacognosy Department, Faculty of Pharmacy, Deraya University, Egypt.

#### Extraction of *Corchorus olitorius* Seeds

One kilogram of dried *Corchorus olitorius* seeds were extracted by maceration in methanol at room temperature three times until exhausted. The alcoholic extract was concentrated under vacuum to yield a viscous syrupy residue (100 g).

### 3.2. Metabolomic Analysis

LCMS was carried out using a Synapt G2 HDMS quadrupole time-of-flight hybrid mass spectrometer (Waters, Milford, MA, USA). The sample (2 μL) was injected into the BEH C18 column, adjusted to 40 °C, and connected to the guard column. Gradient elution of mobile phase was used, starting from no solvent A and 0.1% formic acid in water to 100% acetonitrile as solvent B. MZmine 2.12 was employed for differential investigation of MS data, followed by converting the raw data into positive and negative files in mzML format with ProteoWizard. The compounds were identified via METLIN and DNP databases.

### 3.3. In Vitro Antioxidant Activity

Hydrogen peroxide scavenging and superoxide radical scavenging activities of *Corchorus olitorius* seed extract are discussed in Supplementary Data in detail [60].

#### In Vivo Wound Healing Activity

Twenty-four adult male New Zealand Dutch strain albino rabbits were used. The wound healing potency of *Corchorus olitorius* *seed extract* was assessed utilizing the excision wound model, which is discussed in Supplementary Data with histological study, and gene expression analysis in detail.

### 3.4. Corchorus olitorius Seeds Buccal Films

#### 3.4.1. Preparation Methodology

Three hydrophilic polymers were dispersed in different concentrations, polyvinyl alcohol (PVA), hydroxypropyl methylcellulose (HPMC), and Eudragit S-100 (95%). Double distilled water was used as the main solvent for these polymers. PVA 2 (*w*/*v* %), HPMC 4 (*w*/*v* %), and Eudragit 1 (*w*/*v* %) were prepared isolated. Then, the prepared dispersions were mixed in seven different ratios. The best combination was chosen for Corchorus extract addition. To these mixtures, 2 mL propylene glycol (PG) was added and mixed well on a magnetic stirrer, at low rpm, for a period of 1 h to obtain a homogenous, clear, bubble-free solution. This solution was then poured into a specially fabricated porcelain circular dish for casting. Films were then dried at room temperature for 24 h and packaged in aluminum packs for further investigations.

#### 3.4.2. Characterization of the Prepared Films

The films were characterized by their organoleptic features, weight variation, thickness, disintegration time, and surface morphology. Film appearance was assessed by visual examination to determine the transparency, color, and homogeneity. Weight variation was investigated by determining the average weight of three films [3,61]. Film thickness was measured using a micrometer screw gauge [2]. pH values were measured on swelled films with a digital pH meter [2].

The disintegration behavior of the prepared films was determined by the aid of the reported method [3]. A sample of 10 mL of the simulated fluid was filled into a beaker on a magnetic stirrer at rpm 50 where the film was then placed. The time required for the films to lose their solid-state affording gel properties was recorded as the disintegration time [62].

The film morphology of the selected film was identified using a scanning electron microscope (SEM) (Perkin Elmer, Buckinghamshire, UK). At 0.3 atmospheric pressure and underflow of argon, a part of the film was shielded with a thin slip of gold (150 Å) for 2 min. The film was then imaged at 15 kv × 200 magnification [1].

#### 3.4.3. Clinical Assessment

The clinical study was an experimental randomized double-blinded study. The patients were enrolled by invitation to share in the study and then distributed into two arms randomized. Volunteers were enrolled in the ENT external clinic at the hospital of Minya University from May 2022 to the end of the same month. The sociodemographic data and clinical evaluation of RAMU were gathered by the specialist in addition to a questionnaire regarding age, gender, and the onset of ulcer. The invited volunteers were enrolled according to specific criteria as follows.

#### 3.4.4. Inclusion and Exclusion Criteria

The volunteers were chosen according to specific criteria as: both sexes were accepted with an age range from 18 to 65 years old. The participants must know how to read and write, accept sharing, and sign the informed consent forms. The participants should present with 1 to 5 aphthous ulcers (of less than 72 h duration) with a size no greater than 5 mm in diameter. In addition, a normal sense of pain, without anesthesia or paresthesia.

Participants with a known history of serious drug hypersensitivities, who were pregnant, lactating, and had concurrent clinical conditions that could pose a health risk to the subjects, were excluded from the study. Patients treated with systemic steroids within 1 month of study entry, nonsteroidal anti-inflammatory drugs, or oral antihistamines one month before the study entry, and systemic antibiotics two weeks prior to study entry were also excluded.

#### 3.4.5. Subjects and Study Design

According to the criteria, 21 participants were enrolled in the study. They were distributed into two groups: control and intervention. The distribution was randomized and double-blind. The ethical approval was reviewed by the Institutional Ethics Committee (IEC)/institutional review board (IRB) of Deraya University, and each participant signed a detailed informed consent form. The clinical trial was registered and reviewed by PRS and had identification number NCT05392842.

The participants of the intervention group were treated with the prepared *Corchorus olitorius* seeds buccal fast dissolving film (CoBFDF) whereas the control group was assigned plain films. The clinical examination and patients’ consultation was carried out and followed up by the ENT physician and the clinical pharmacist. The clinical examinations included the size of ulcers, inflammation area, and pain. Ulcer size and inflammation were assessed by the investigator, while the pain was evaluated using the visual analog scale (VAS method) that was determined by the patient [63]. The pain score, inflammation, and size of ulcers were assessed at zero time before film application and daily for six days. The evaluations were obtained under a 12 h fasting condition.

Pain scoring by VAS comprised a 10 cm straight line between ends, with 0 representing no pain (better outcome) and 10 for intolerable pain (worse outcome). Subjects were asked to mark the point that best represented the present pain level of the ulcer. The ulcer size was determined by a calibrated probe and estimated as the diameter of the white border. The degree of erythema was evaluated by the investigators on a 4-point scale ranging from 0 to 3 [64] 0, No erythema, 1, light red/pink, 2, red but not dark in color, 3, very red, dark [65].

The efficacy indices (EI) for ulcer pain, size, and erythema were calculated using the following formula.

(1) $$\mathrm{EI}=\frac{v_{f}-v_{i}}{v_{f}}\times 100$$

v_f_ refers to values measured on the fourth day and v_i_ refers to the zero time. The evaluation of efficacy indices (EI) was as follows; EI ≥ 95% for healing, EI ≥ 70% to <95% for remarkable improvement, EI ≥ 30% to <70% for moderate improvement, and EI < 30% for no improvement [66].

### 3.5. In Silico Molecular Modeling

Wound healing activity of 21 dereplicated compounds from *Corchorus olitorius* seed was carried out using the X-ray crystals of *TNF-α*, *IL-1β*, and GSK protein structures PDB 2AZ5, 6Y8M, and 1Q5K, respectively, and is discussed in the Supplementary Data in detail.

## 4. Conclusions

In this study, *Corchorus olitorius* seed extract displayed remarkable wound healing activity in the treatment of recurrent minor aphthous ulceration (RMAU) by accelerating wound closure rate, enhancing *TGF-β*, as well as *TNF-α*, expression, and suppressing inflammatory markers (*TNF-α*, and *IL-1β*). Twenty-one compounds were dereplicated and identified from *C. olitorius* seeds. Molecular docking analysis on *TNF-α*, *IL-1β*, and *GSK3* predicted the possible mode of action in the wound healing activity. Furthermore, the potent in vitro antioxidant activity of *Corchorus olitorius* seed extract that is attributed to its SOD and H_2_O_2_ scavenging activity could eliminate ROS and hence enhance its wound-healing activity. Finally, this study recommended the application of *C. olitorius* seed extract in wound care as a promising therapy to accelerate the wound healing process; however, future detailed mechanistic studies are still required to confirm those results.

## Figures and Tables

**Table 1:** Dereplicated compounds from *Corchorus olitorius* seeds.

No	Name	Structure	Source	Exact Mass	Ref.
**1**	Corchorifatty acid C.	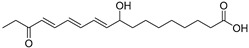	*Corchorus olitorius*	308.417	[23]
**2**	Digitoxigenin boivinoside.	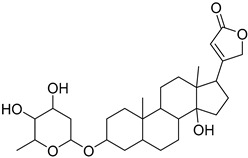	*Corchorus olitorius*	504.662	[24]
**3**	4,7-Dihydroxycoumarin.	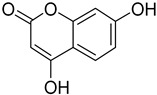	*Corchorus olitorius*	178.02661	[25]
**4**	9-Hydroxy-10-undecenoic acid	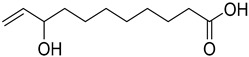	*Corchorus olitorius*	200.141245	[23]
**5**	Corchorifatty acid B.	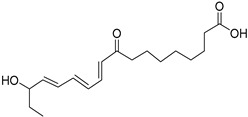	*Corchorus olitorius*	308.19876	[23]
**6**	Corchorifatty acid F.	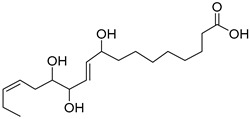	*Corchorus olitorius*	328.224975	[23]
**7**	Corchoionoside C.	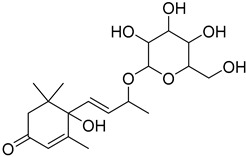	*Corchorus olitorius*	386.19407	[26]
**8**	Corchoionoside A.	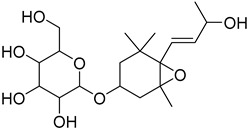	*Corchorus olitorius*	388.20972	[26]
**9**	Corchoionoside B.	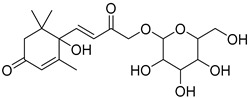	*Corchorus olitorius*	400.173335	[26]
**10**	Corchoroside B.	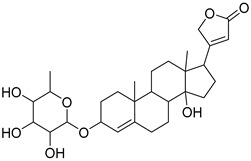	*Corchorus olitorius*	518.28797	[24]
**11**	Trachelosperogenin A.	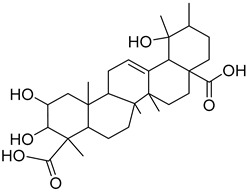	*Corchorus olitorius*	518.324355	[28]
**12**	Corchoroside A.	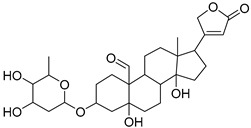	*Corchorus olitorius*	534.282885	[29]
**13**	Corchorosol A.	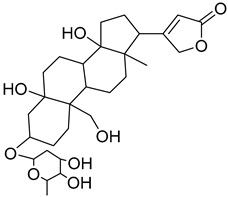	*Corchorus olitorius*	536.298535	[24]
**14**	Corchoruside B.	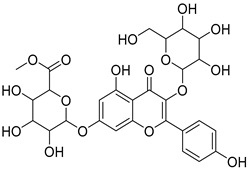	*Corchorus olitorius*	638.148305	[30]
**15**	Coroloside.	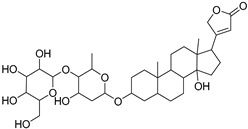	*Corchorus olitorius*	666.36153	[12]
**16**	Corchorusoside B.	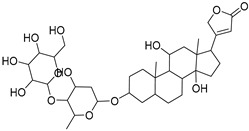	*Corchorus olitorius*	682.356445	[31]
**17**	Olitoriside.	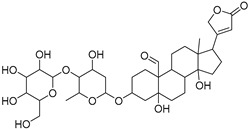	*Corchorus olitorius*	696.33571	[32]
**18**	Corchorusoside D.	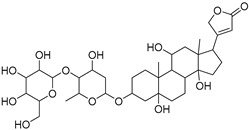	*Corchorus olitorius*	698.35136	[31]
**19**	Corchorusoside A	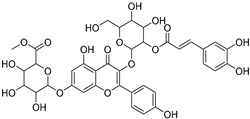	*Corchorus olitorius*	800.10	[31]
**20**	Olitoriusin.	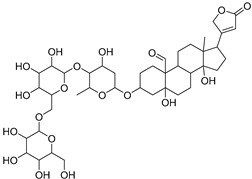	*Corchorus olitorius*	858.388535	[32]
**21**	Corchorusoside E.	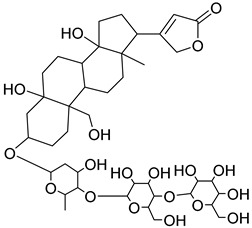	*Corchorus olitorius*	860.404185	[31]

**Table 2:** Sociodemographic analysis of participants.

Sociodemographic Characteristics	Control Group	Intervention Group	*p*-Value
Age	36.6 ± 6	35.6 ± 4	0.74
Sex (male %)	45 ± 2	55 ± 3	0.7
Residence (rural %)	50 ± 0.5	50 ± 1	<0.5
Education (well-educated %)	55 ± 0.3	45 ± 0.2	<0.5
Onset of ulcers (days)	1 ± 0.3	1 ± 0.6	<0.5

**Table 3:** Clinical assessment of human volunteer’s average at baseline of treatment.

Observation	Control Group	Intervention Group	*p*-Value
Pain score (0–10)	8 ± 1	7 ± 2	0.74
Ulcer size (mm)	5 ± 2	5 ± 3	0.71
Erythema (0–5)	3 ± 0.5	4 ± 1	0.81

**Table 4:** Clinical assessment of human volunteers on the fourth day.

Observation	Control Group	Intervention Group	*p*-Value
Pain score	6.5 ± 0.5	4 ± 2	0.042 *
Ulcer size (mm)	3 ± 1	0 ± 2	0.0052 **
Erythema	2 ± 0.5	1 ± 1.5	0.034 *

* Means there are a significant difference between the control and intervention group (*p* ≤ 0.05), ** means there are a significant difference between the control and intervention group *p* ≤ 0.01.

**Table 5:** Efficacy incidences of the intervention group at the end of treatment.

Observation	Control Group	Intervention Group
Pain score (EI%)	45%	75%
Ulcer size (EI%)	25%	70%
Erythema (EI%)	50%	75%

**Table 6:** The molecular docking results of compounds **1**–**21** using PDB 2AZ5 and 6Y8M for *TNF-α* and *IL-1β*, respectively.

Compound	*TNF-α* (PDB 2AZ5)				*IL-1β* (PDB 6Y8M)			
	Binding Energy Score (kcal/mol)	Interacting Residues	Interaction Type	Interaction Distance (Å)	Binding Energy Score (kcal/mol)	Interacting Residues	Interaction Type	Interaction Distance (Å)
**Co-crystallized ligand**	**−7.16**	Leu120	H-donor	3.02	**−4.77**	Met44	H-donor	4.27
		Leu120	pi-H	4.69		Met148	H-donor	4.13
		Tyr59	pi-pi	3.94		Lys103	H-acceptor	3.01
						Lys103	H-acceptor	3.24
						Lys103	H-acceptor	2.97
						Leu110	pi-H	4.45
**1**	**−6.27**	Gly122	H-acceptor	3.35	**−6.20**	Met148	H-donor	3.65
		His15	H-pi	4.09		Arg11	H-acceptor	2.94
		Tyr59	H-pi	4.44		Arg11	H-acceptor	3.42
		Tyr59	H-pi	4.21		Lys103	H-acceptor	3.04
						Thr147	H-acceptor	3.41
**2**	**−6.97**	**Tyr119**	H-pi	4.38	**−7.49**	Asn53	H-donor	3.25
						Gln149	H-acceptor	3.23
						Lys103	H-acceptor	3.08
**3**	**−4.26**	**Tyr119**	H-donor	3.08	**−4.09**	Thr147	pi-H	4.90
		Tyr151	H-acceptor	3.61		Met148	pi-H	4.10
		Gln61	H-acceptor	3.69		Met148	pi-H	4.37
		Tyr151	H-acceptor	3.54				
**4**	**−5.20**	**Tyr119**	H-donor	3.08	**−5.52**	Met148	H-acceptor	2.93
		Tyr151	H-acceptor	3.61		Thr147	H-acceptor	3.27
		Gln61	H-acceptor	3.69		Met148	H-acceptor	3.28
		Tyr151	H-acceptor	3.54				
**5**	**−6.46**	Tyr151	H-acceptor	3.14	**−7.13**	Met148	H-acceptor	3.53
		Leu120	H-acceptor	3.52		Arg11	H-acceptor	3.09
		Gly121	H-acceptor	3.01		Arg	ionic	2.93
						Arg	ionic	3.55
**6**	**−6.39**	Lys98	H-acceptor	3.25	**−6.79**	Lys103	H-acceptor	2.72
		Ile118	H-acceptor	3.6		Asp54	H-acceptor	3.30
		**Tyr119**	H-acceptor	3.71		Met148	H-acceptor	3.23
		Lys98	H-acceptor	3.28				
		Lys98	ionic	3.37				
**7**	**−6.33**	**Tyr119**	H-donor	3.31	**−7.76**	Asn53	H-donor	3.08
		Gly121	H-donor	3.08		Thr147	H-acceptor	3.29
						Met148	H-acceptor	3.09
						Lys103	H-acceptor	2.94
**8**	**−6.34**	**Tyr119**	H-donor	3.15	**−6.95**	Asn53	H-donor	3.05
		Leu120	H-acceptor	3.47		Asn108	H-donor	3.18
		Gly121	H-acceptor	3.35		Lys103	H-acceptor	2.98
						Met148	H-acceptor	3.35
**9**	**−6.33**	Lys98	H-acceptor	3.24	**−7.22**	Met148	H-donor	3.19
		**Tyr119**	H-pi	4.63		Asn53	H-donor	2.88
						Lys103	H-acceptor	3.50
						Met148	H-acceptor	3.51
						Lys103	H-acceptor	2.86
**10**	**−7.38**	**Tyr119**	H-donor	3.51	**−7.37**	Met148	H-donor	3.39
		**Tyr119**	H-pi	4.35		Gln15	H-donor	3.09
		Tyr151	H-pi	5		Met148	H-acceptor	3.12
		**Tyr119**	H-pi	4.64		Gln149	H-acceptor	3.50
						Arg11	H-acceptor	3.01
**11**	**−5.72**	Tyr151	H-acceptor	3.11	**−6.16**	Met148	H-donor	2.88
		Tyr59	H-pi	4.25		Thr147	H-acceptor	3.37
						Lys103	ionic	2.74
**12**	**−6.63**	Gly121	H-donor	3.46	**−8.01**	Met148	H-donor	3.40
		Gly121	H-acceptor	3.17		Met148	H-acceptor	2.93
		Tyr59	H-pi	4.74		Gln149	H-acceptor	3.07
		Tyr59	H-pi	3.77				
**13**	**−6.81**	Leu120	H-donor	2.97	**−7.52**	Asn108	H-donor	2.81
		**Tyr119**	H-donor	3.2		Met148	H-acceptor	3.17
		Tyr59	H-pi	4.48		Lys103	H-acceptor	2.96
		Tyr59	H-pi	4.68		Gln149	H-acceptor	3.18
**14**	**−7.75**	Tyr151	H-acceptor	3.12	**−8.44**	Asn53	H-donor	2.89
		**Tyr119**	pi-H	3.53		Asn108	H-donor	3.03
		**Tyr119**	pi-H	4.33		Lys103	H-donor	2.96
		**Tyr119**	pi-H	4.75		Thr147	H-acceptor	3.35
						Asn108	H-acceptor	3.33
						Asp54	H-acceptor	3.68
						Gln149	H-acceptor	3.32
**15**	**−7.81**	Gly121	H-donor	3.04	**−8.63**	Met148	H-donor	3.47
		Tyr151	H-acceptor	2.96		Asn53	H-donor	2.91
		Tyr151	H-acceptor	3.01		Asn53	H-donor	3.22
		Gly121	H-acceptor	3.08		Asp54	H-donor	3.56
		**Tyr119**	H-pi	4.52		Glu105	H-donor	3.38
		Tyr59	H-pi	3.66		Gln149	H-acceptor	3.21
**16**	**−7.61**	Gly148	H-donor	3.02	**−8.34**	Met148	H-donor	3.05
		Asn34	H-donor	2.91		Met148	H-donor	3.65
		Gln149	H-acceptor	3.27		Asp54	H-donor	2.89
		Tyr59	H-pi	3.64		Thr147	H-acceptor	3.49
		His15	H-pi	4.38		Lys103	H-acceptor	2.97
		Tyr151	H-pi	4.07		Lys103	H-acceptor	3.05
**17**	**−8.36**	Gln61	H-donor	3.15	**−8.87**	Met148	H-donor	3.61
		Gln149	H-donor	2.92		Met148	H-acceptor	2.90
		**Tyr119**	H-pi	4.66		Gln149	H-acceptor	3.58
		Tyr59	H-pi	4.93		Arg11	H-acceptor	3.53
						Gln149	H-acceptor	3.42
						Arg11	H-acceptor	3.10
**18**	**−8.44**	Gly121	H-donor	2.9	**−8.49**	Asn53	H-donor	3.00
		Ser95	H-donor	3.38		Glu105	H-donor	3.29
		Gly121	H-donor	3.42		Gln149	H-acceptor	3.53
		Ser95	H-donor	3.54		Lys103	H-acceptor	3.13
		Tyr151	H-acceptor	3.54		Lys103	H-acceptor	2.97
		Tyr151	H-acceptor	2.96				
**19**	**−9.37**	Ser95	H-donor	2.87	**−9.72**	Glu105	H-donor	3.34
		Leu120	pi-H	4.95		Asp54	H-donor	2.76
		Gly121	pi-H	4.77		Met148	H-donor	3.06
		Gly121	pi-H	4.82		Lys103	H-acceptor	2.94
						Phe46	H-pi	3.86
						Met148	H-pi	4.64
**20**	**−8.83**	Glu146	H-donor	3.47	**−9.17**	Asn108	H-donor	2.87
		Glu146	H-donor	3.12		Met148	H-donor	2.90
		Gly121	H-donor	3.64		Met148	H-acceptor	3.19
		**Tyr119**	H-pi	3.84		Arg11	H-acceptor	3.17
		Tyr115	H-pi	4.35		Gln149	H-acceptor	3.58
**21**	**−9.08**	Glu146	H-donor	2.84	**−9.74**	Met148	H-donor	3.49
		Glu146	H-donor	2.79		Asn108	H-donor	2.89
		Tyr151	H-acceptor	2.91		Asn108	H-donor	3.19
		**Tyr119**	H-pi	4.53		Met148	H-donor	2.94
						Ser152	H-acceptor	3.00
						Lys103	H-acceptor	3.20
						Lys103	H-acceptor	2.89
						Glu105	H-acceptor	3.46
						Lys103	H-acceptor	3.09

**Table 7:** The molecular docking results of compounds **1**–**21** using PDB 1Q5K for *GSK3* enzyme.

Cpd	Binding Energy Score (kcal/mol)	Interacting Residues	Interaction Type	Interaction Distance (Å)
TMU	**−8.20**	VAL135	H-donor	2.49
		ASP133	H-donor	3.35
		VAL135	H-donor	3.11
		VAL135	H-acceptor	3.22
		ILE62	pi-H	3.89
**1**	**−8.59**	PRO136	H-donor	2.77
		LYS60	H-acceptor	2.99
		GLN72	H-acceptor	3.45
		LYS60	H-acceptor	2.88
		LYS60	ionic	2.99
**2**	**−8.32**	LYS85	H-acceptor	3.75
		ARG144	H-acceptor	2.99
		ARG144	H-acceptor	3.35
**3**	**−5.39**	VAL135	H-donor	3.33
		ASP200	H-acceptor	3.22
		VAL70	pi-H	4.29
		VAL70	pi-H	4.59
**4**	**−7.33**	PRO136	H-donor	2.92
		ASP200	H-acceptor	3.24
		LYS85	H-acceptor	3.57
**5**	**−7.99**	CYS199	H-donor	3.76
		LYS60	H-acceptor	2.97
		LYS85	H-acceptor	3.59
		LYS60	H-acceptor	2.99
		GLN72	H-acceptor	3.52
		LYS60	ionic	2.97
**6**	**−8.97**	ARG141	H-acceptor	3.14
		ARG144	H-acceptor	2.94
		TYR134	H-acceptor	3.35
		ARG144	H-acceptor	3.02
		VAL135	H-acceptor	3.24
**7**	**−8.40**	VAL135	H-donor	3.11
		ILE62	H-acceptor	3.58
**8**	**−8.81**	CYS199	H-donor	3.67
		ILE62	H-donor	3.26
		PRO136	H-donor	3.19
		LYS85	H-acceptor	3.53
**9**	**−8.36**	CYS199	H-donor	3.52
		ARG141	H-acceptor	3.41
		ARG144	H-acceptor	3.07
		ARG144	H-acceptor	3.1
		LYS85	H-acceptor	3.12
		TYR140	H-pi	4.97
**10**	**−8.14**	GLN185	H-donor	3.16
		VAL135	H-donor	3.1
		ARG141	H-acceptor	3.34
**11**	**−7.70**	ARG141	H-acceptor	3.06
		LYS85	H-acceptor	2.8
		ARG144	ionic	3.98
**12**	**−8.66**	CYS199	H-donor	3.33
		GLN185	H-donor	3.39
		GLN185	H-donor	3.47
		CYS199	H-donor	3.62
**13**	**−9.04**	ASN186	H-donor	3.44
		ILE62	H-donor	3.39
		VAL61	H-donor	3.39
		PHE67	H-acceptor	3.3
**14**	**−11.13**	CYS199	H-donor	3.8
		CYS199	H-donor	4.29
		PRO136	H-donor	2.8
		ILE62	H-donor	3.13
		CYS199	H-acceptor	3.17
		ASP200	H-acceptor	3.15
		ILE62	pi-H	3.93
		ARG141	pi-H	3.52
**15**	**−10.37**	ILE62	H-donor	3.67
		ARG141	H-acceptor	3.12
**16**	**−11.49**	ASN186	H-donor	3.91
		ASP200	H-donor	2.82
		PRO136	H-donor	3.51
		ARG141	H-acceptor	3.21
**17**	**−10.12**	ILE62	H-donor	3.26
		VAL61	H-donor	2.97
		ARG144	H-acceptor	3.27
		ARG141	H-acceptor	3.08
		LYS85	H-acceptor	3.64
**18**	**−9.92**	ILE62	H-donor	3.41
		CYS199	H-donor	3.67
		ILE62	H-acceptor	3.52
		ARG141	H-acceptor	3.35
		ASP200	H-acceptor	3.5
		LYS85	H-acceptor	3.07
**19**	**−11.29**	ASN64	H-donor	3.3
		GLN72	H-acceptor	3.13
		ILE62	pi-H	3.82
		VAL70	pi-H	4.22
		ARG141	pi-H	4.23
		CYS199	pi-H	3.69
**20**	**−11.45**	ILE62	H-donor	3.37
		ILE62	H-donor	3.54
		GLN185	H-donor	3.69
		CYS199	H-donor	3.51
		ILE62	H-acceptor	3.54
		CYS199	H-acceptor	3.68
		ASP200	H-acceptor	3.13
**21**	**−9.71**	ILE62	H-donor	3.24
		LYS60	H-acceptor	3.19
		ARG141	H-acceptor	3.36
		LYS183	H-acceptor	3.33

## Data Availability

The data presented in this study are available on request from the corresponding authors.

## Supplementary Materials

The following supporting information can be downloaded at: https://www.mdpi.com/article/10.3390/molecules27207020/s1, The potential of *Corchorus olitorius* seeds buccal films for treatment of recurrent minor aphthous ulcerations in human volunteers [37,60,67,68,69,70,71,72].
