# Supplementary material for: The Potential of Corchorus olitorius Seeds Buccal Films for Treatment of Recurrent Minor Aphthous Ulcerations in Human Volunteers
Source: Molecules. 2022 Oct 18;27(20):7020. doi: 10.3390/molecules27207020 (PMC9610436; doi:10.3390/molecules27207020)
Supplement: Supplementary file 1 [file molecules-27-07020-s001.zip › molecules-1929998-supplementary.pdf]

Supplementary Materials

The Potential of *Corchorus olitorius* Seeds Buccal Films for Treatment of Recurrent Minor Aphthous Ulcerations in Human Volunteers

Nourhan Hisham Shady [1,*], Abdullah H. Altemani [2], Faisal H. Altemani [3], Sherif A. Maher [4,5], Mahmoud A. Elrehany [4], Entesar Ali Saber [6,†], Ahmed M. Badawi [7], Fatma Mohamed Abd El-Mordy [8], Nada M. Mohamed [9], Mohammed A. S. Abourehab [10,11,*], Ahmed M. Sayed [12], Usama Ramadan Abdelmohsen [1,13,*] and Soad A. Mohamad [14]

[1] Department of Pharmacognosy, Faculty of Pharmacy, Deraya University, Universities Zone, New Minia City 61111, Egypt
[2] Department of Family and Community Medicine, Faculty of Medicine, University of Tabuk, Tabuk 71491, Saudi Arabia
[3] Department of Medical Laboratory Technology, Faculty of Applied Medical Sciences, University of Tabuk, Tabuk 71491, Saudi Arabia
[4] Department of Biochemistry, Faculty of Pharmacy, Deraya University, Universities Zone, New Minia City 61111, Egypt
[5] Department of Biochemistry, Faculty of Pharmacy, New Valley University, El Kharga 71511, Egypt
[6] Department of Histology and Cell Biology, Faculty of Medicine, Minia University, Minia 61519, Egypt
[7] Department of Otorhinolaryngology, Faculty of Medicine, Minia University, Minia 61519, Egypt
[8] Department of Pharmacognosy and Medicinal Plants, Faculty of Pharmacy (Girls), Al-Azhar University, Cairo 11754, Egypt
[9] Department of Pharmaceutical Chemistry, Faculty of Pharmacy, Modern University for Technology, and Information (MTI), Cairo 11754, Egypt
[10] Department of Pharmaceutics, College of Pharmacy, Umm Al-Qura University, Makkah 21955, Saudi Arabia
[11] Department of Pharmaceutics and Industrial Pharmacy, College of Pharmacy, Minia University, Minia 61519, Egypt
[12] Department of Pharmacognosy, Faculty of Pharmacy, Nahda University, Beni-Suef 62513, Egypt
[13] Department of Pharmacognosy, Faculty of Pharmacy, Minia University, Minia 61519, Egypt
[14] Department of Pharmaceutics and Clinical Pharmacy, Faculty of Pharmacy, Deraya University, Universities Zone, New Minia City 61111, Egypt
* Correspondence: norhan.shady@deraya.edu.eg (N.H.S.); maabourehab@uqu.edu.sa (M.A.S.A.); usama.ramadan@mu.edu.eg (U.R.A.); Tel.: +20-86-2347759 (U.R.A.)
† Current address: Delegated to Deraya University, Universities Zone, New Minia City 61111, Egypt

S1. Materials and Methods

S1.1. Quantitative Real-Time Polymerase Chain Reaction (qRT-PCR)

Approximately 50 mg of dorsal skin tissues was homogenized using a ultrasonic homogenizer (SFX 550 Branson Digital Sonifier® ultrasonic cell disruptor/homogenizer is versatile. Danbury, CT, USA) in 0.5 mL of TRIzol TM reagent (Amresco, Solon, OH, USA). RNA was extracted from gastric mucosa tissue using the TRIzol TM RNA Extraction Reagent (Amresco, Solon, OH, USA) as instructed by the manufacturer. The overall RNA concentration was estimated at A260 nm, and the purity was measured based on the ratio A260/A280. Samples with purity $\geq$ 1.7 were used for qRT-PCR. GAPDH was used as a reference housekeeping gene. cDNA synthesis was performed for equivalent amounts of total RNA in all samples using the RevertAid H Minus First Strand cDNA Synthesis Kit (#K1632, Thermo Science Fermentas, St. Leon-Ro, Germany) as directed by the manufacturer. Real-time PCR was conducted with single-stranded cDNAs. The sequences of the used primers are shown in (Table S1) PCR reactions were conducted by SYBER Green (#K0251, Thermo Scientific Fermentas St. Leon-Ro, Germany-Maxima SYBER Green qPCR Master Mix (2X)) using a StepOne Real-Time PCR Detection System (Applied Biosystems).

Table S1. Primer sequence of the genes included in the study.

Gene name	GenBank accession		
IL-1β	NC_013670.1	Forward	5'-AGCTTCTCCAGAGCCACAAC-3'
		Reverse	5'-CCTGACTACCCTCACGCACC-3'
GAPDH	NC_013676.1	Forward	5'-GTCAAGGCTGAGAACGGGAA-3'
		Reverse	5'-ACAAGAGAGTTGGCTGGGTG-3'
TGF-β1	NC_013672.1	Forward	5'-GACTGTGCGTTTTGGGTTCC-3'
		Reverse	5'-CCTGGGCTCCTCCTAGAGTT-3'
TNF-α	NC_013680.1	Forward	5'-GAGAACCCCACGGCTAGATG-3'
		Reverse	5'-TTCTCCAACTGGAAGACGCC-3'

The real-time polymerase chain reaction (qRT-PCR) was achieved using 20 µl of RealMOD Green qRT-PCR Mix kit (iNtRON biotechnology) with 0.02 µg RNA per reaction and 10 Pmol of unique primers, for 30 cycles of 95 °C for 10 s. and 60 °C for 1 min. The comparative Ct (threshold cycle) approach was used to assess the relative concentrations of the products. The relative expression was determined using formula $2(-^{\Delta\Delta Ct})$ [1]. They were scaled compared to the control where control samples were set at a value of 1.

S1.2. In Vitro Antioxidant Activity

S1.2.1. Hydrogen Peroxide Scavenging Activity

The reaction with a defined amount of exogenously provided H_2O_2 was used to determine the hydrogen peroxide (H_2O_2) scavenging activity that reflects the antioxidative capacity of *Corchorus olitorius* seed extract. Colorimetric analysis was used to estimate the residual H_2O_2 [2, 3] [4]. In brief, 20µl of the extract was mixed with 500 µl of H_2O_2 and incubated at 37ºC for 10 minutes. After that, 500 µl of enzyme/3, 5-dichloro-2-hydroxylbenzenesulfonate solution was added and incubated at 37ºC for 5 minutes. Colorimetrically, the intensity of the colored product was measured at 510 nm. The positive control was ascorbic acid. The percentage of H_2O_2 scavenging activity was determined by comparing the results of the test with those of the control using the following formula:

$$\text{scavenging activity} = \frac{\text{A control} - \text{A sample}}{\text{A control}} \times 100 \quad \text{(S1)}$$

The IC_{50} of each sample was calculated after performing the assay at four different concentrations (1000 µg/mL, 500 µg/mL, 250 µg/mL, and 125 µg/mL) using Graph pad prism 7 software.

S1.2.2. Superoxide Radical Scavenging Activity

The superoxide anion scavenging activity was measured as described by Sreenivasan et al. [5, 6]. The superoxide anion radicals were formed in a Tris–HCl buffer (16 mM, pH8.0) containing 90 µl of NBT (0.3 mM), 90 µL of NADH (0.936 mM), 0.1mL of *Corchorus olitorius* seed extract (125, 250, 500, and 1000 g/mL), and 0.8 mL Tris–HCl buffer (16 mM, PH 8.0). The reaction was initiated by adding 0.1 mL phenazine methosulfate (PMS) solution (0.12 mM) to the mixture, which was then incubated at 25˚C for 5 minutes, and at 560 nm, the absorbance was measured. Ascorbic acid was selected as a reference. The percentage inhibition was obtained by comparing the test findings to those of the control using the formula below:

$$\text{Superoxide scavenging activity} = \frac{\text{A control} - \text{A sample}}{\text{A control}} \times 100 \quad \text{(S2)}$$

IC_{50} was calculated using Graph pad prism 7 software by performing the test at four different concentrations.

S1.2.3. In silico molecular modeling

X-ray crystals of protein structures PDB 2AZ5, 6Y8M, and 1Q5K for *TNF-α, IL-1β*, and GSK3, respectively, were downloaded from the Protein Data Bank, corrected and 3D-protonated at cutoff 15 Å using amber 10:EHT forcefield of the Molecular Operating Environment (MOE 2014.0901) software. The best quality chains were selected to perform the molecular docking followed by water molecule removal. Their binding sites were selected using their corresponding co-crystallized ligand atoms at a grid of 4.5 Å followed by docking protocol validation using redocking of the co-crystallized ligands until achieving the lowest RMSD with the highest binding energy score. The molecular docking process was performed using triangle matcher, London dG, GBVI/WSA as the placement, rescoring function 1 and 2, respectively, for the three protein structures. The tested compounds **1-21** were chemically drawn using Chemdraw Ultra 12.0 then transferred as smiles to the MOE builder window, with their hydrogens added and energy minimized at the same forcefield.

S1.3. In vivo Wound-Healing Activity

S1.3.1. Animal Treatment and Ethical Statement

This study involved twenty-four adult male New Zealand Dutch strain albino rabbits. Throughout the experiment, the rabbits were placed in polypropylene cages with unlimited access to a natural pellet diet and water ad libitum. Seven days before the study's commencement, the animals were placed in well-ventilated animal houses with normal laboratory locations including temperature (25 ± 2°C), relative humidity (44–56%), as well as day and dark cycles (12:12h.). The Ethics Committee at the Faculty of Pharmacy, Deraya University authorized this study and stated that animals should not suffer at any stage of testing and should be kept in line with the Guide for the Care and Use of Laboratory Animals (ethical permission No: 10/2021).

S1.3.2. Preparation of the Test Samples for the Bioassay, Circular Excision Wound Mode, and Experimental Design

The rabbits were anesthetized using an intraperitoneal (I.P.) injection with ketamine (Alphasam company®, Holland, 50 mg/kg) and xylazine hydrochloride (Alphasam company®, Holland, 10 mg/kg [7]. After anesthesia, the awareness (alertness) level of the rabbits was determined and shaving was carried out. The shaving area was the back of the animal, in the withers. Anticipation was performed by alcohol 70% and povidone–iodine 10% 7 times. The animals were depilated on the paravertebral area before wound creation and a circular excision wound of 6 mm in diameter was created using a biopsy punch [8]. This procedure generates the wound in both the epidermis and the dermis layers. Using experimental rabbits, 3 groups were formed; each group included 8 rabbits. The rabbits of **Group 1** did not receive any treatment (bare wound) and were used as the negative control. Wounds in **Group 2** were treated with *Corchorus olitorius seeds extract* (2 mg/wound), while **Group 3** was treated with MEBO® ointment (100 mg/wound) and used as positive control (market treatment). The wounded area was covered with a standard surgical dressing while redressing was performed with fresh dressing at 3, 7, and 10 days.

S1.3.3. Collection of Tissue Samples and Percentage of Wound Closure Rate

For the physical appearance and closure of wounds, photographs of wounded areas were taken using a digital camera (DSC-W320 Sony; Sony Corp., Tokyo, Japan) at 0, 3, 7, 10, and 14 days, posing vertically to the middle of wound with a distance of 6 cm. The reduction in the wounded area (wound closure) was used as an indicator of the efficacy of the treatment. Thus, the periphery of the excisional wound was outlined after creating the wound with the help of transparent paper. On days 7 and 14, full-thickness skin biopsies of entire ulcers from all groups were collected under anesthesia. The closure in wounds was recorded at 3, 7, 10, and 14 days and expressed as a percent of the healed wounded area. The wound area was evaluated using Image J 1.49v software (National

Institutes of Health, Bethesda, MD, USA), and the wound closure rate was calculated as a percentage change in the original wound area using the following formula:

$$\text{Wound closure (\%)} = \frac{\text{Area of wound on day 0} - \text{Area of wound on day nth}}{\text{Area of wound on day 0}} \times 100 \quad \text{(S3)}$$

Where n represents the order of the examination day, i.e., 3rd, 7th, 10th, and 14th.

Finally, the day of complete wound healing (epithelialization) of each wound was observed for all 3 groups.

S1.3.4. Histological Study

Rabbits were anesthetized on the 7th and 14th days and the wounded area with a periphery of about 5 mm of ambient unwounded dorsal skin biopsy was taken. These skin tissues were fixed in a 10 % formalin solution for 2-3 days followed by tissue processing by handling through a graded list of alcohol and xylene then embedding in paraffin blocks. Thin tissue sections of 5 μm thickness were made using Microtome and stained with hematoxylin and eosin stain (H&E). The stained sections were observed using a light microscope fitted with a camera in terms of neovascularization, epidermis, scar, granulation tissues, and density of collagen fibers, and images were taken using the Leica Application Suite (Leica Microsystems, Wetzlar, a light microscope).

S1.4. Data Statistical Analysis

The statistical analysis was performed using GraphPad Prism (LaJolla, CA). The Shapiro–Wilk test for normality of variance and then nonlinear fit of normalized variables were performed. Leven's test for homogeneity of variance was performed and, finally, a two-way ANOVA was performed. The results were represented as mean ±standard deviation (SD). Two-way ANOVA was applied to determine whether the results had significant variations and a *P*-value ≤ 0.001 was considered significant.

References

1. VanGuilder, H.D.; Vrana, K.E.; Freeman, W.M. Twenty-five years of quantitative PCR for gene expression analysis. *Biotechniques* **2008**, *44*, 619–626.
2. Hassan, H.A.; Abdel-Aziz, A.F. Evaluation of free radical-scavenging and anti-oxidant properties of black berry against fluoride toxicity in rats. *Food Chem. Toxicol.* **2010**, *48*, 1999–2004.
3. Musa, A.; Shady, N.; Ahmed, S.; Alnusaire, T.; Sayed, A.; Alowaiesh, B.; Sabouni, I.; Al-Sanea, M.; Mostafa, E.; Youssif, K.; et al. Antiulcer potential of *Olea europea* L. Cv. arbequina leaf extract supported by metabolic profiling and molecular docking. *Antioxidants* **2021**, *10*, 644.
4. Shady, N.H.; Soltane, R.; Maher, S.A.; Saber, E.A.; Elrehany, M.A.; Mostafa, Y.A.; Sayed, A.M.; Abdelmohsen, U.R. Wound Healing and Antioxidant Capabilities of Zizyphus mauritiana Fruits: In-Vitro, In-Vivo, and Molecular Modeling Study. *Plants* **2022**, *11*, 1392.
5. Sonboli, A.; Mojarrad, M.; Ebrahimi, S.N.; Enayat, S. Free radical scavenging activity and total phenolic content of methanolic extracts from male inflorescence of Salix aegyptiaca grown in Iran. *Iran J. Pharm. Res.* **2010**, *9*, 293–296.
6. Alsenani, F.; Ashour, A.M.; Alzubaidi, M.A.; Azmy, A.F.; Hetta, M.H.; Abu-Baih, D.H.; Elrehany,M.A.; Zayed, A.; Sayed, A.M.; Abdelmohsen, U.R.; et al. Wound Healing Metabolites from Peters' Elephant-Nose Fish Oil: An In Vivo Investigation Supported by In Vitro and In Silico Studies. *Mar. Drugs* **2021**, *19*, 605.
7. Ozbilgin, S.; Ozkardesler, S.; Akan, M.; Boztas, N.; Ozbilgin, M.; Ergur, B.U.; Derici, S.; Guneli, M.E.; Meseri, R. Renal ischemia/reperfusion injury in diabetic rats: the role of local ischemic preconditioning. *BioMed Res. Int.* **2016**, *2016*, 8580475.
8. Sandhu, S.K.; Kumar, S.; Raut, J.; Singh, M.; Kaur, S.; Sharma, G.; Roldan, T.L.; Trehan, S.; Holloway, J.; Gabriella Wahler, G.; et al. Systematic development and characterization of novel, high drug-loaded, photostable, cur-cumin solid lipid nanoparticle hydrogel for wound healing. *Antioxidants* **2021**, *10*, 725.
